# Supplementary material for: COVID-19 medical papers have fewer women first authors than expected
Source: eLife. 2020 Jun 15;9:e58807. doi: 10.7554/eLife.58807 (PMC7304994; doi:10.7554/eLife.58807)
Supplement: Supplementary file 1. [file elife-58807-supp1.docx]

**Supplementary file 1**

**Details of the mixed regression models used to estimate the adjusted means and 95% confidence limits shown in Figure 1.**

***Mixed logit model with first-author gender as outcome (woman=1).***

|  |  | **Coef.** | **SE (robust)** | **95% CI** |  |
| --- | --- | --- | --- | --- | --- |
| **Fixed** | Intervention (dummy) | -.336 | .079 | -.492 | -.181 |
| **Random** | Variance intercept | .337 | .580 |  |  |
|  | Variance coefficient | .205 | .453 |  |  |
|  | Number of disciplines | 160 |  |  |  |
|  | Number of observations | 23,469 |  |  |  |
|  | Log Likelihood | -14881.7 |  |  |  |

***Mixed logit model with last-author gender as outcome (woman=1).***

|  |  | **Coef.** | **SE (robust)** | **95% CI** |  |
| --- | --- | --- | --- | --- | --- |
| **Fixed** | Intervention (dummy) | -.117 | .086 | -.286 | .052 |
| **Random** | Variance intercept | .446 | .668 |  |  |
|  | Variance coefficient | .243 | .493 |  |  |
|  | Number of disciplines | 161 |  |  |  |
|  | Number of observations | 24,175 |  |  |  |
|  | Log Likelihood | -13581.9 |  |  |  |

***Mixed Logit Model Predicting the overall share of women per article as outcome.***

|  |  | **Coef.** | **SE (robust)** | **95% CI** |  |
| --- | --- | --- | --- | --- | --- |
| **Fixed** | Intervention (dummy) | -.08 | .047 | -.175 | -.010 |
| **Random** | Variance intercept | .292 | .541 |  |  |
|  | Variance coefficient | .133 | .365 |  |  |
|  | Number of disciplines | 166 |  |  |  |
|  | Number of observations | 21,119 |  |  |  |
|  | Log Likelihood | -31660.2 |  |  |  |

***Mixed logit model with first-author gender as outcome (woman=1) (Time factor analysis).***

|  |  | **Coef.** | **SE (robust)** | **95% CI** |  |
| --- | --- | --- | --- | --- | --- |
| **Fixed** | March+April (Dummy) | -.424 | .092 | -.604 | -.244 |
|  | May (Dummy) | -.223 | .108 | -.435 | -.010 |
| **Random** | Variance intercept | .338 | .581 |  |  |
|  | Variance coefficient (March+April) | .082 | .287 |  |  |
|  | Variance coefficient (May) | .253 | .503 |  |  |
|  | Number of disciplines | 160 |  |  |  |
|  | Number of observations | 23,469 |  |  |  |
|  | Log Likelihood | -14880.5 |  |  |  |

***Mixed logit model with last-author gender as outcome (woman=1) (Time factor analysis).***

|  |  | **Coef.** | **SE (robust)** | **95% CI** |  |
| --- | --- | --- | --- | --- | --- |
| **Fixed** | March+April (Dummy) | -.213 | .110 | -.429 | .003 |
|  | May (Dummy) | -.028 | .111 | -.246 | .189 |
| **Random** | Variance intercept | .442 | .665 |  |  |
|  | Variance coefficient (March+April) | .1885 | .434 |  |  |
|  | Variance coefficient (May) | .234 | 0.484 |  |  |
|  | Number of disciplines | 161 |  |  |  |
|  | Number of observations | 24,175 |  |  |  |
|  | Log Likelihood | -13580.5 |  |  |  |

***Mixed Logit Model Predicting the overall share of women per article as outcome (Time factor analysis).***

|  |  | **Coef.** | **SE (robust)** | **95% CI** |  |
| --- | --- | --- | --- | --- | --- |
| **Fixed** | March+April (Dummy) | -.197 | .443 | -.301 | -.053 |
|  | May (Dummy) | .130 | .361 | -.120 | .104 |
| **Random** | Variance intercept | .293 | .541 |  |  |
|  | Variance coefficient (March+April) | .197 | .443 |  |  |
|  | Variance coefficient (May) | .130 | .361 |  |  |
|  | Number of disciplines | 166 |  |  |  |
|  | Number of observations | 21,119 |  |  |  |
|  | Log Likelihood | -31654.9 |  |  |  |
